# Low bentonite biomass leads to inconsistent culture-based estimates of microbial abundances

**DOI:** 10.1093/femsle/fnag003

**Published:** 2026-01-13

**Authors:** Rachel C Beaver, Cailyn M Perry, Chang Seok Kim, Josh D Neufeld

**Affiliations:** Department of Biology, University of Waterloo, 200 University Avenue West, Waterloo, Ontario N2L 3G1, Canada; Department of Biology, University of Waterloo, 200 University Avenue West, Waterloo, Ontario N2L 3G1, Canada; Nuclear Waste Management Organization (NWMO), Toronto, Ontario M4T 2S3, Canada; Department of Biology, University of Waterloo, 200 University Avenue West, Waterloo, Ontario N2L 3G1, Canada

**Keywords:** bentonite, heterogeneity, cultivation, DNA sequencing, clay, deep geological repository

## Abstract

Bentonite is an important component of deep geological repositories for long-term storage of used nuclear fuel. Studying the microbiology of bentonite exposed to various conditions is relevant because certain microorganisms (e.g. those that produce corrosive sulfide or gaseous metabolites) could lead to deterioration of engineered barrier components of the repository. In previous research, a high degree of variability in the abundance of culturable microorganisms among replicate samples has been observed. The purpose of this study was to test whether experimental technique (e.g. inadequate mixing of bentonite) or extremely low biomass represent mechanisms to explain such variability. Using a combination of cultivation- and DNA-based techniques to study six replicate hydrated bentonite microcosms, as well as six replicate bentonite aliquots originating from the same hydrated bentonite microcosm, the results of this study demonstrate that observed heterogeneity is likely not due to inadequate bentonite mixing. Instead, the data indicate that low biomass of as-received bentonite leads to unique populations of culturable bacteria associating with each sample, or to a lesser degree within different areas of a single bentonite sample used to establish a microcosm. Because some microorganisms that grow in bentonite are culturable under commonly used cultivation conditions and others are not, this can lead to differences in culture-based abundance estimates among replicate samples. Although cultivation is a useful technique to demonstrate viability of microorganisms in bentonite, the results of this study highlight the importance of a multifaceted experimental approach (i.e. coupling cultivation to DNA-based analysis) and careful analysis of replicates when working with such low biomass samples.

## Teaser

High level of heterogeneity in abundance of culturable microorganisms previously reported in bentonite is likely due to low biomass effects.

## Introduction

Bentonite is a key component of deep geological repository (DGR) designs for the long-term storage of used nuclear fuel. Due to the long timeframe a DGR must remain functional (i.e. between 100 000 and 1 million years), and the importance of maintaining separation between used nuclear fuel and the surrounding environment, safety must be considered from multiple perspectives (NWMO 2024). In the case of microbiology, certain microorganisms could negatively impact a DGR by contributing to microbiologically influenced corrosion of metal engineered barrier components or by production of gases, which in sufficient volume could create permeability fissures in the bentonite (Boylan et al. 2019). A broad goal of this area of research is to identify the abundance and composition of any microbial communities present in bentonite under various DGR-relevant conditions.

As-received bentonite (i.e. relatively dry, “as-received” from the manufacturer) has a low abundance of culturable microorganisms and microbial DNA (Vachon et al. 2021). This low biomass can occasionally lead to observed heterogeneity in both abundance of culturable microorganisms and microbial community profiles (Vachon et al. 2021). When bentonite is hydrated, microorganisms often increase in abundance, assuming other conditions (e.g. swelling pressure and temperature) are favorable for their growth or revival from a state of desiccation (Stroes-Gascoyne et al. 2010, 2011, Jalique et al. 2016, Burzan et al. 2022, Engel et al. 2023, Beaver et al. 2024, 2025b, Punch et al. 2025). In these previous experiments, the abundance of culturable microorganisms often resulted in plate counts with “countable” numbers of colonies (i.e. ∼20–200), which would be expected to result in similar abundance estimates among replicates. However, a high degree of variability, especially in the abundance of culturable aerobic heterotrophs, was still sometimes observed for replicates (Stroes-Gascoyne 2010, Stroes-Gascoyne et al. 2010, Jalique et al. 2016, Engel et al. 2023, Beaver et al. 2024, 2025a, b, Punch et al. 2025). For early timepoints of pressure vessel incubations where bentonite is not presaturated, this variability can be explained by uneven saturation leading to distinct swelling pressures and water activities throughout the clay plug (Beaver et al. 2024, 2025b). For other experiments, especially microcosms established with hydrated bentonite that is thoroughly mixed before incubation, it is unclear whether differences in the abundance of culturable aerobic heterotrophs for biological replicates are due to actual microbial heterogeneity in the bentonite or potentially inconsistent experimental technique. Further, it is challenging to identify outliers when experiments are limited to duplicate or triplicate measurements (Beaver et al. 2025a, Punch et al. 2025). In either case, it can be challenging to make predictions about the potential abundance of microorganisms in a future DGR when there is inconsistency among replicates, and potential inherent microbial heterogeneity of as-received bentonite is poorly understood. This issue goes well beyond lab-based concerns and is a problem for global studies that rely on robust bentonite abundance estimates for identifying the suitability of engineered barrier conditions for a DGR.

The goal of this study was to investigate the extent and origins of heterogeneity in abundance of culturable microorganisms in bentonite, a topic that has been noted but never systematically examined. Specifically, we tested whether heterogeneity arises both among and within replicate bentonite microcosms. Additionally, we tested if heterogeneity reflects true biological variation, or is instead an experimental artifact, by comparing different bentonite–water mixing methods. Although previous studies have reported variability in microbial abundance under differing saturation, oxygen, and temperature conditions, none have attempted to eliminate confounding environmental factors to explicitly test whether bentonite itself remains heterogeneous under controlled and nominally homogeneous conditions. Here, we selected an experimental design that intentionally minimizes heterogeneity-inducing gradients by manually mixing bentonite and water thoroughly, hydrating only small amounts of bentonite, and incubating samples in Mason jars with ample oxic headspace to avoid locally anoxic zones.

A DGR is expected to experience a wide range of temperature, oxygen, and saturation conditions, making many sets of conditions “DGR relevant” (Guo 2016, King et al. 2017). We selected experimental conditions that fit within the realm of DGR relevant (i.e. 45°C, oxic, and high water activity), but that have also been shown previously to result in both an increase in microbial abundance after hydration and incubation, which is necessary for observing heterogeneity in microbial growth and variability between replicates (Beaver et al. 2025a). In a DGR, bentonite will be compacted to a range of dry densities, and many laboratory studies accordingly use compacted bentonite to simulate DGR pressure conditions (Stroes-Gascoyne et al. 2010, 2011, Jalique et al. 2016, Burzan et al. 2022, Engel et al. 2023, Beaver et al. 2025a, b). Other studies intentionally use uncompacted bentonite to isolate the influence of individual factors, such as water activity and temperature, on bentonite microbial communities, independent of the effects of compaction (Beaver et al. 2025a, Punch et al. 2025). Heterogeneity in microbial abundance has been observed across both compacted and uncompacted studies. Here, we selected an uncompacted experimental design to avoid additional variability introduced by dry density gradients and to allow for sufficient microbial growth, which is restricted at higher dry densities, to exceed limits of detection for cultivation-based quantification.

To capture variability more comprehensively than previous studies, we coupled cultivation-based quantification to DNA sequencing analysis of both bentonite and culture biomass, using a larger number of replicates than previously included for bentonite experiments. This design allowed us to examine differences in total culturable heterotroph abundance in addition to differences in the identity of heterotrophs that emerge across replicate microcosms. We hypothesized that heterogeneity originates from the very low initial cell numbers in as-received bentonite, such that even after thorough mixing, individual aliquots may contain distinct microbial taxa and therefore give rise to divergent growth trajectories.

## Methods

To assess sources of variability in abundance of culturable aerobic heterotrophs among and within replicate hydrated bentonite experiments, a series of hydrated bentonite Mason jar microcosms were established. All microcosms were prepared on the laboratory bench under oxic conditions using 10 g of a single batch of Wyoming MX-80 bentonite (referred to throughout as MX9) hydrated with 3 ml of ultrapure (Type I) water (resistivity >18 MΩ/cm at 25°C) to achieve a bentonite water activity of 0.99. All microcosms were incubated under oxic conditions for 1 week at 45°C, because these are conditions both previously observed to have a high variability among biological replicates (Beaver et al. 2025a), and within the range of expected temperature and oxygen conditions for a DGR (Guo 2016, King et al. 2017). Oxic headspace conditions were ensured throughout incubation by placing an anaerobic indicator strip (BD) on the inside of the lid of each Mason jar.

**Figure 1:**
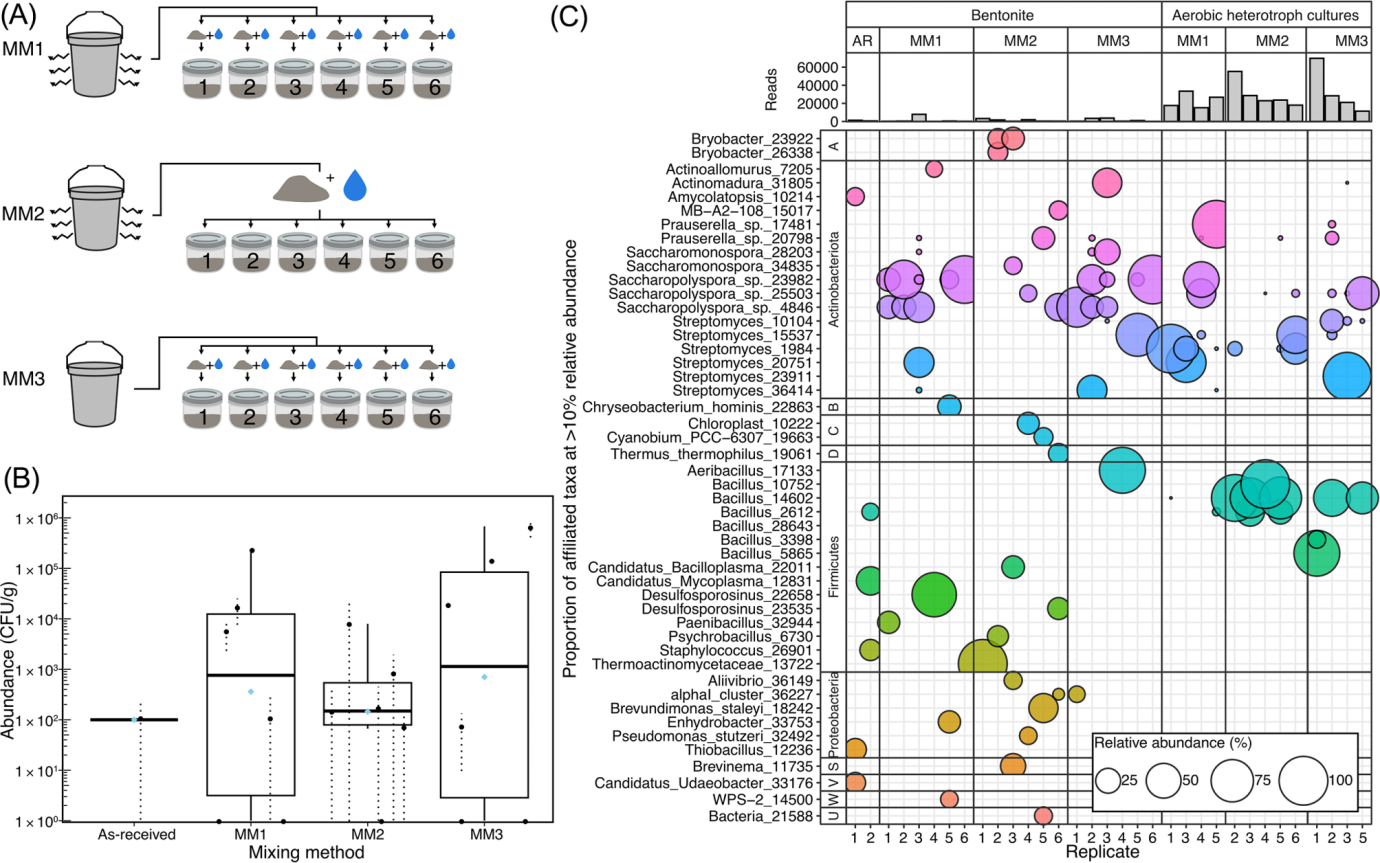
Schematic of experimental setup (A), abundance of culturable aerobic heterotrophs in six replicate microcosms set up using mixing methods (MM) 1, 2, and 3 (Table 1; B), and corresponding 16S rRNA gene profiles of bentonite from each microcosm as well as of as-received bentonite (AR) and biomass from the aerobic heterotroph cultures inoculated with each bentonite sample, with associated sequencing read counts (C). Error bars in bar plots represent the standard deviation in abundance of culturable heterotrophs on three replicate agar plates. Only genera with a minimum relative abundance of 10% in at least one sample are included in the bubble plot. Additional phylum name letter designations include Acidobacteriota (A) Bacteroidota (B), Cyanobacteria (C), Deinococcota (D), Spirochaetota (S), Verrucomicrobiota (V), WPS-2 (W), and unclassified (U).

In the first experiment, six replicate hydrated bentonite microcosms were established using each of three methods of mixing bentonite and water (Table 1, Fig. 1A) to both test variability between microcosms, and to test if insufficient mixing increases heterogeneity. Mixing method one was used in previous microcosm studies and involved mixing the as-received bentonite stock, then hydrating an aliquot of bentonite separately for each microcosm (Beaver et al. 2025a, Punch et al. 2025). Method two would be expected to potentially generate more consistent replicates, as the bentonite for all six replicates was hydrated together and then split into six different microcosms. Method three was the same as method one, but the as-received bentonite stock bucket was not mixed prior to collecting bentonite. After mixing, hydrated clay was added to sterile 125 ml Mason jars. From each microcosm, one aliquot of bentonite was collected after 1 week of incubation at 45°C for each of cultivation (2 g) and DNA analysis (50 mg). Subsequently, to test for variability in the abundance of culturable aerobic heterotrophs within microcosms, a new set of triplicate microcosms was established using mixing method one (Table 1, Fig. 3A). From each, six aliquots of bentonite were collected for each of cultivation and DNA analysis after 1 week of incubation at 45°C. Aerobic heterotrophs were cultured on Reasoner’s 2A agar (R2A; M1687, HiMedia Laboratories) and incubated for 5 days at 30°C using methods previously described (Beaver et al. 2025a).

**Table 1:** Methods of hydrating and mixing bentonite for microcosms setup.

Mixing method	Description
1	As-received stock bentonite mixed by agitation, then six individual bentonite aliquots weighed and hydrated separately.
2	As-received stock bentonite mixed by agitation, then bentonite for six microcosms was weighed, hydrated, then separated into six separate microcosms.
3	As-received stock bentonite not mixed. Six individual bentonite aliquots weighed and hydrated separately.

After incubation of cultures, biomass was collected from agar plates using a sterile swab (Puritan). Genomic DNA was extracted from clay samples using the DNeasy PowerSoil kit (Qiagen) and from culture biomass using the DNeasy UltraClean kit (Qiagen). For both kits, the manufacturer’s instructions were followed, except for the lysis and final elution steps. After addition of lysis solution, PowerBead tubes were incubated at 70°C for 10 min, followed by bead beating for 45 s at 5.5 m/s using a FastPrep-24 Classic Instrument (MP). To concentrate DNA, final elution volumes of 60 and 50 µl were used for PowerSoil and UltraClean extractions, respectively. The V4–V5 region of the 16S rRNA gene was amplified from each genomic DNA extract of bentonite and culture biomass and sequenced on a MiSeq System (Illumina). Sequencing and sequence analysis included negative DNA extraction kit controls and polymerase chain reaction (PCR) no template controls. Sequenced negative controls were used to identify and remove potential contaminants using the Decontam R package (Davis et al. 2018). Sequencing and sequence analysis methods are described in detail elsewhere (Beaver et al. 2025a). Sequence data was deposited to the European Nucleotide Archive with accession number PRJEB79491.

An ANOVA was used to test for differences in abundance of culturable microorganisms among treatments using a *P*-value threshold of .05. Each sample group included the six replicate log-transformed culture counts. Prior to calculating Bray–Curtis distances, samples were normalized to 5000 reads using the Scaling with Ranked Subsampling package in R (Beule and Karlovsky 2020, Heidrich et al. 2021).

## Results and discussion

### Heterogeneity among replicate microcosms

The first goal of this study was to explore heterogeneity in the abundance of culturable aerobic heterotrophs among replicate microcosms. As previously observed (Stroes-Gascoyne 2010, Stroes-Gascoyne et al. 2010, Jalique et al. 2016, Engel et al. 2023, Beaver et al. 2024, 2025a, b, Punch et al. 2025), the abundance of culturable microorganisms in the present study was variable (Fig. 1). For several microcosms, counts of culturable aerobic heterotrophs were in the order of 10^5^, and in others they were not detected at all (Fig. 1B). The average abundance of culturable heterotrophs was approximately two orders of magnitude lower than those reported for a 30°C experiment with a similar design (Punch et al. 2025), and one order of magnitude lower than those observed in a previous experiment performed under identical conditions, though variability between replicates was very high in the latter experiment (Beaver et al. 2025a).

To evaluate whether variation could be reduced or exacerbated by more (method two) or less (method three) thorough mixing of the bentonite starting material used for replicate microcosms, three different methods of mixing as-received bentonite with water were tested (Table 1). All three mixing methods produced replicate microcosms with similarly variable abundances of culturable aerobic heterotrophs (Fig. 1B). Standard deviations were proportionally similar across mixing methods (equal to between 1.9 and 2.2 times the average), and the average abundance of culturable heterotrophs for replicates of each mixing method were not significantly different (ANOVA, *P* = .82). Variability in abundance of culturable aerobic heterotrophs was smaller among technical replicate agar plates than among biological replicate microcosms, although when fewer than 20 colonies were counted on replicate plates, standard deviation between technical replicates also appeared relatively large (Fig. 1B). These results indicate that observed heterogeneity in abundance of culturable microorganisms was not an artifact of insufficient or inconsistent bentonite mixing during microcosm preparation; instead, heterogeneity appears to be an inherent property of hydrated bentonite under these conditions.

Our experiment demonstrates the broader experimental challenge well. The six microcosms of each mixing condition, and arguably all 18 microcosms in the experiment, were prepared to be identical and should, in principle, have yielded similar microbial abundances. Yet, the wide range of values measured demonstrates that typical experimental design, often relying on duplicate or triplicate samples, could easily lead to erroneous conclusions. For example, if only three replicates were picked at random from the present experiment, the conclusion could be that microbial abundance relative to the starting material increased, decreased, remained unchanged, or was too variable to interpret, depending on the replicates selected.

In addition to variability in culturable abundances of aerobic heterotrophs, 16S rRNA gene profiles were also variable among microcosms (Fig. 1C). Sequencing depth was lower for microcosms established using mixing method two, potentially explaining the more variable profiles between replicates. Bentonite samples from microcosms were most commonly dominated by sequences associated with *Saccharopolyspora* (Fig. 1C), which have been previously observed to dominate uncompacted hydrated bentonite microcosms (Beaver et al. 2025a, Punch et al. 2025). However, several individual microcosms had little or no representation of these genera in their 16S rRNA gene profiles and were instead dominated by sequences associated with representatives of phyla *Actinobacteria, Firmicutes*, or *Proteobacteria*.

**Figure 2:**
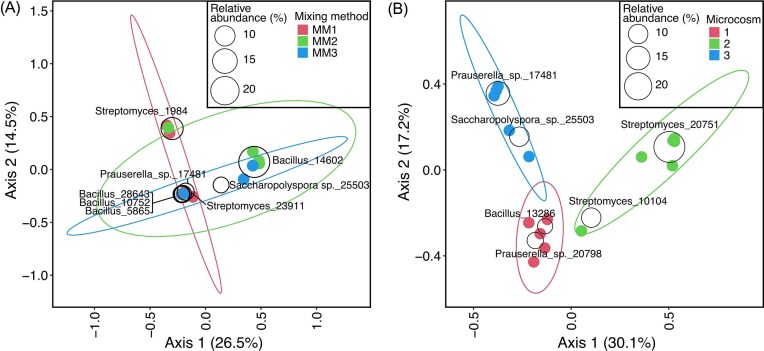
Principal coordinate analysis plots of aerobic heterotroph culture samples from experiment 1 (A) and 2 (B), based on Bray–Curtis distances. ASVs with a minimum relative abundance of 5% are displayed as clear circles. Ellipses represent 95% confidence intervals around the centroid of the sample group.

Although infrequently detected in bentonite profiles, the most common taxon dominating 16S rRNA gene profiles of cultures from the bentonite was affiliated with the *Bacillus* genus, though once again there was variability among replicate microcosms, and several microcosms were instead dominated by sequences associated with other taxa such as *Streptomyces* (Fig. 1C). In ordination space, the profiles of culturable aerobic heterotrophs did not group based on mixing method, supporting cultivation results that also demonstrated that insufficient mixing is likely not the cause of variability in community composition (Fig. 2A).

Comparing bentonite and culture profiles as well as abundance results provides insight into the cause of heterogeneity in abundance. For several microcosms, abundant ASVs in the bentonite were also detected in the associated cultures. For example, bentonite from replicate three established using mixing method one had high relative abundance of an ASV associated with *Streptomyces* (20 751), and this same ASV dominated the culture biomass profile for the same sample, demonstrating that the species that grew in the bentonite was culturable under the conditions employed. In contrast, sequences associated with *Saccharopolyspora* often dominated the 16S rRNA gene profiles of hydrated bentonite after incubation (Fig. 1B) and were detected in low or no abundance for as-received MX9, suggesting that *Saccharopolyspora*-associated cell division occurred in the microcosms. However, this taxon was rarely detected in the 16S rRNA gene profiles of associated cultures (Fig. 1B), suggesting that it may not be culturable under the conditions applied. This taxon also dominated the profiles of previously conducted microcosm experiments, and similarly was not detected in 16S rRNA gene profiles of associated cultures (Punch et al. 2025). As an even more extreme example, *Thermoactinomycetaceae* dominated the microbial profile of bentonite from replicate one established using mixing method two (i.e. 3184 of the 3187 total reads), suggesting that members of this family grew in the bentonite. However, the corresponding culture had no detectable culturable aerobic heterotrophs, even though members of family *Thermoactinomycetaceae* are typically aerobic heterotrophs (Jiang et al. 2019). This taxon was also previously found to dominate microbial profiles of bentonite from microcosms, but not those of the associated cultures (Beaver et al. 2025a), further suggesting that it may not be culturable under the selected conditions. A similar observation was made with a different thermophile in a study on Bavarian bentonite, where no microbial activity was detected despite a shift in community composition towards *Caldinitratiruptor microaerophilus*-dominated communities (Matschiavelli et al. 2019). Broadly, for every replicate where no growth was detected via cultivation, there was a shift in community composition relative to the as-received bentonite, suggesting growth, and results suggest that dominant taxa in these samples are likely not culturable under the applied conditions, explaining why no growth was detected via cultivation.

### Heterogeneity within replicate microcosms

**Figure 3:**
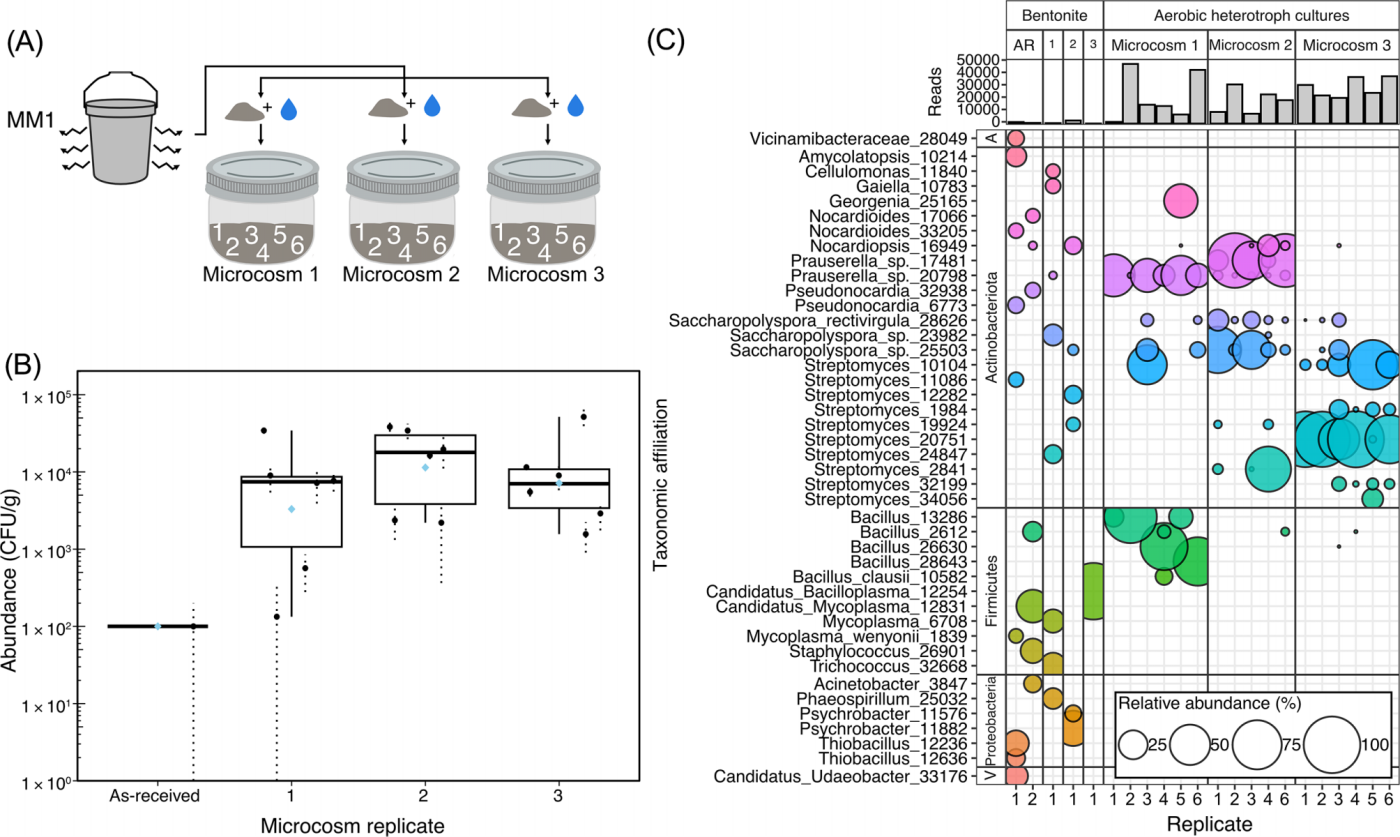
Schematic of experimental setup (A), abundance of culturable aerobic heterotrophs in six subsamples of three replicate microcosms (B), and corresponding 16S rRNA gene profiles of bentonite from each of the three microcosms as well as of as-received bentonite (AR) and biomass from aerobic heterotroph cultures, with associated sequencing read counts (C). Error bars in bar plots represent the standard deviation in abundance of culturable heterotrophs on three replicate agar plates. Only genera with a minimum relative abundance of 5% in at least one sample are included in the bubble plot. Additional phylum name letter designations include *Acidobacteriota* (A) and *Verrucomicrobiota* (V).

Because variation was observed among replicate microcosms, a second experiment was conducted to test if a similar level of variation would be observed if sets of cultures were inoculated using different aliquots of bentonite originating from the same microcosm. To test this, a total of three new microcosms were established using mixing method one. After microcosm incubation, six separate aliquots of bentonite were collected from each microcosm for cultivation (Table 1, Fig. 3A). Though to a lesser degree, there was still observed variability in the abundance of culturable aerobic heterotrophs in each set of cultures (Fig. 3B). Despite variation, the average abundances of culturable aerobic heterotrophs were not significantly different for all three microcosms (ANOVA, *P* = .41).

The 16S rRNA gene profiles associated with the second experiment were variable (Fig. 3C). In general, 16S rRNA gene profiles of aerobic heterotroph cultures inoculated with aliquots of hydrated bentonite from the same microcosm were dominated by similar sequences (Fig. 3C), but this was not always the case. For example, three replicate bentonite samples from microcosm one had aerobic heterotroph cultures dominated by sequences associated with *Bacillus*, whereas three replicate bentonite samples taken from the same microcosm instead had aerobic heterotroph cultures with high relative abundances of sequences associated with *Prauserella, Georgenia, Saccharopolyspora*, and *Streptomyces* (Fig. 3C). Similarly, most cultures inoculated with bentonite from microcosm two were dominated by sequences associated with *Prauserella*, but others instead by sequences associated with *Streptomyces* or *Saccharopolyspora* (Fig. 3C). Cultures inoculated with bentonite from microcosm 3 had the most consistent 16S rRNA gene profiles, all dominated by sequences associated with *Streptomyces* (Fig. 3C). Nonetheless, profiles of culturable aerobic heterotrophs grouped by microcosm in ordination space (Fig. 2B), demonstrating that within-microcosm variability in community composition is lower than among-microcosm variability.

The average abundance of culturable microorganisms in the within-microcosm experiment (Fig. 3B) was approximately an order of magnitude greater than that measured in the initial among-microcosm experiment (Fig. 1B) for microcosms prepared with the same mixing method (method one), despite both experiments using identical materials and methods. Further, none of the replicates in the within-microcosm experiment had zero culturable microorganisms. This contrast further illustrates the experimental challenge revealed in the first experiment. Although the within-microcosm experiment contained 18 samples (i.e. the same as the initial experiment), these represent only three distinct microcosms. It seems likely that by chance the three microcosms happened to include microbial communities dominated by microorganisms that were readily culturable under the applied conditions. If considered in isolation, one might conclude that there was growth in the bentonite microcosms in the range of two orders of magnitude. However, the results of the first experiment indicate that these results may not be reproducible. Based on the level of heterogeneity observed among microcosms, it is reasonable to predict that if, for example, three additional independent microcosms had been included in this second experiment, at least one would have no detectable culturable heterotrophs, which would decrease the overall average abundance estimate.

## Conclusions and recommendations

The results of this study demonstrate heterogeneity in the abundance and identity of culturable aerobic heterotrophs, both among replicate microcosms and within individual microcosms, regardless of the bentonite water mixing method, and despite conditions intentionally designed to minimize heterogeneity. Abundances of culturable heterotrophs varied by orders of magnitude and included instances of no detectable culturable microorganisms, despite apparent shifts in community composition relative to the as-received bentonite. The results demonstrate that heterogeneity is not attributable to insufficient bentonite mixing during microcosm preparation, but rather reflects underlying heterogeneity in the microbial communities themselves.

One explanation for the observed heterogeneity is that the very low initial abundance of cells in as-received bentonite leads to stochastic and uneven distributions of microorganisms among and within microcosms, such that different species are differentially represented in certain microcosms or, to a lesser degree, areas within a microcosm. When bentonite is hydrated, the specific organisms that happen to be present in a given microcosm may proliferate, but only a subset will be culturable under the conditions applied. As a result, microcosms with similar total microbial abundances may nonetheless exhibit stark differences in culturable counts, reflecting differences in community composition, and specifically the proportion of the total community that is culturable, rather than differences in overall growth. Overall, heterogeneity likely represents the combined effects of low initial biomass, leading to stochastic representation of taxa in each microcosm, and only a portion of bentonite microorganisms being culturable under the chosen conditions.

This study highlights the importance of replicates, a multifaceted experimental approach (e.g. cultivation coupled to DNA sequencing), and cautious interpretation to obtain a representative view of the microbial community abundance and composition in bentonite. Future bentonite microbiology studies should incorporate at least triplicates, and potentially more replicates to initially explore heterogeneity of an experimental system. Although replication will not remove heterogeneity, it can help to demonstrate the nature of the variability (e.g. whether there is a single outlier due to experimental technique or whether the heterogeneity is likely inherent to the sample). It is also important to include a variety of types of replicates (e.g. multiple samples from the same microcosm, and also separate replicate microcosms). The inclusion of numerous replicates in this study revealed that a variety of different microorganisms can proliferate in bentonite and influence cultivation-based estimates to varying degrees. Under other more limiting DGR-relevant conditions, it is possible that fewer groups of microorganisms will be capable of growth, which may reduce between-sample heterogeneity, though depending on which microorganisms can grow, cultivation-based abundance estimates may still be impacted.

Statistical tests remain valuable for identifying differences in microbial abundance between treatments, but variability among replicates can obscure true differences. In some contexts, using a less stringent significance threshold (e.g. *P* < .10 rather than < .05) can reduce the risk of type II errors when the consequences of overlooking microbial growth are more severe than those of falsely detecting growth (e.g. if a statistical test incorrectly indicates microbial growth under a given condition, a type I error, the conclusion would be to recommend more stringent DGR conditions than actually necessary, which is a better scenario than the opposite).

Statistical tests aside, discussions of these types of samples should also explicitly acknowledge and address the role of heterogeneity in the interpretation of data. This is made possible by use of a multifaceted experimental approach (e.g. cultivation coupled to DNA-based analyses), which can help better reflect the underlying microbial distribution within such low biomass samples. For example, if no growth is observed using cultivation, DNA-based evidence of community composition shifts can either corroborate or challenge that conclusion. Although this study focused on aerobic heterotroph growth under oxic, high-water activity, and relatively high-temperature microcosm conditions, similar low-biomass-driven heterogeneity is likely to affect other microbial groups that grow under different bentonite incubation conditions. Consequently, heterogeneity remains an important consideration for studies exploring the microbiology of bentonite.

## Notes

Repositories: All sequences were submitted to the European Nucleotide archive with accession number PRJEB79491.
